# Squamous Cell Carcinoma Originating from a Dermoid Cyst Located between the Upper Rectum and Prostate: A Case Report

**DOI:** 10.70352/scrj.cr.25-0724

**Published:** 2026-01-28

**Authors:** Takao Tsukahara, Hiroyuki Takahashi, Mikiko Aoki, Yosuke Chuman, Issei Takeshita, Keiichi Shiokawa, Taro Munechika, Hideki Nagano, Yoshiko Matsumoto, Ken Nagata, Makoto Hamasaki, Suguru Hasegawa

**Affiliations:** 1 Department of Gastroenterological Surgery, Fukuoka University Hospital, Fukuoka, Fukuoka, Japan; 2 Department of Pathology, Fukuoka University Hospital, Fukuoka, Fukuoka, Japan

**Keywords:** squamous cell carcinoma, dermoid cyst, rectum, robot assisted surgery

## Abstract

**INTRODUCTION:**

Pelvic squamous cell carcinoma (SCC) originating from a dermoid cyst (DC) is extremely rare. Here, we report the first case of SCC that developed in the anterior rectal area.

**CASE PRESENTATION:**

A 46-year-old man was initially diagnosed with rectal SCC by endoscopic biopsy, and multimodal image findings demonstrated direct invasion of the urinary system. Accordingly, total pelvic exenteration with bilateral lymph node dissection was performed with robotic assistance, and the patient was discharged without severe postoperative complications. Histological assessment revealed that the cancer originated in a DC with direct invasion of the seminal vesicle.

**CONCLUSIONS:**

Pelvic DC–derived SCC is rare but possesses high malignant potential. Because of the difficulty in preoperative diagnosis, diagnostic excision may be selected if complete resection is possible. Importantly, robot-assisted surgery enables precise management and might be an optimal strategy for preventing cancer dissemination by rupture during surgery.

## Abbreviations

CRT
: chemoradiation therapy
DC
: dermoid cyst
SCC
: squamous cell carcinoma
TPE
: total pelvic exenteration

## INTRODUCTION

Developmental abnormalities during the fetal period include DCs, which are developmental cysts that sometimes arise in the cerebellum and presacral space.^1)^ The incidence of malignant DCs is extremely rare. Here, we report the first case of SCC that originated from an anterior rectal DC, and the achievement of a radical cure by minimum invasive surgery under robotic assistance.

## CASE PRESENTATION

A 46-year-old man consulted a primary care doctor with the chief complaints of melena and pneumaturia. After colonoscopy, he was diagnosed with rectal cancer and referred to our institution. Intriguingly, a pathological assessment revealed SCC, a rare histological type.^2)^ He had no previous medical or surgical history. Upon physical examination, his abdomen was soft and flat, without tenderness.

**Fig. 1:**
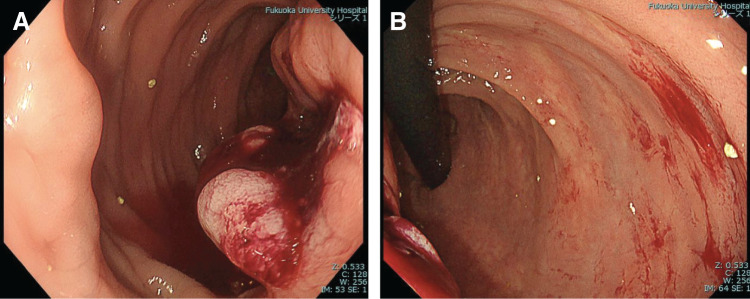
Colorectal endoscopic finding. (**A**) Colorectal examination shows an advanced tumor with deep ulceration on the left-sided upper rectum. No obvious fistula is seen on the mucosal surface. (**B**) Tumor is located at approximately 6 cm proximal from anal verge.

Blood tests showed slightly high levels of SCC-related antigen (3.2 ng/mL) and the levels of other tumor markers including carcinoembryonic antigen and carbohydrate antigen 19-9 were all within the normal range. Colorectal examination showed an advanced tumor with deep ulceration on the upper rectum, with no obvious fistula on the mucosal surface (**Fig. 1A** and **1B**).

**Fig. 2:**
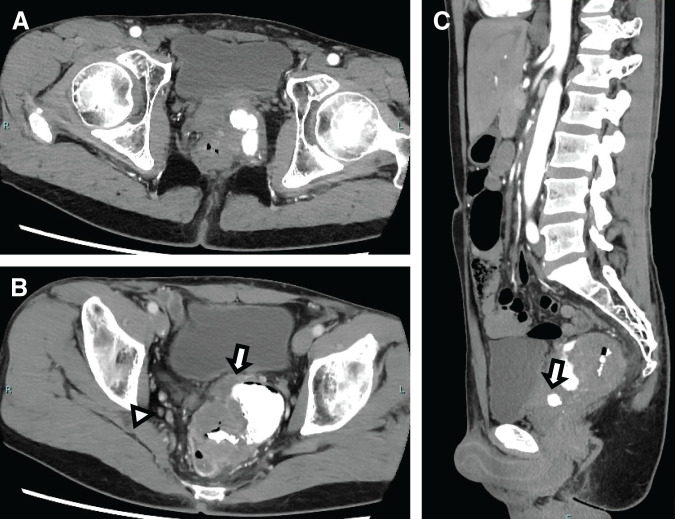
CT scan findings. (**A**) Horizontal image. There is a lobulation-like tumor on the left pelvic cavity with density of calcification and air. (**B**) Horizontal image. The tumor connects to the prostate, indicating direct invasion (arrow). Right-sided lateral lymph node shows a swelling (arrowhead). (**C**) Sagittal image. The mass also contacts with the urethra in the prostate, indicating fistulation (arrow).

**Fig. 3:**
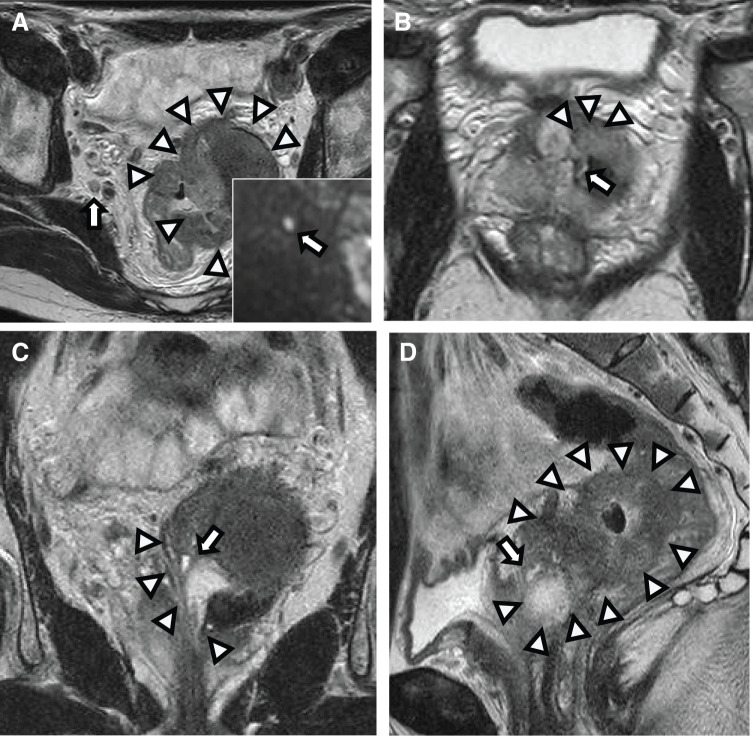
MRI findings. (**A**) T2-wighted, horizontal image. There is a lobulation-like tumor on the left pelvic cavity with hypointense inside. The right-sided lateral lymph node also shows hypointense (arrowheads). The lower right image indicates a diffusion-weighted, horizontal image. The right-sided lateral lymph node also shows hyperintense (arrow). (**B**) T2-wighted, horizontal image. The tumor connects to the prostate, invading the inner gland beyond the outer lesion (arrowheads). Arrow may indicate fistulation. (**C**) T2-wighted, coronal image. The tumor connects to the urinary system (arrowheads). Arrow may indicate fistulation. (**D**) T2-wighted, sagittal image. The tumor widely spreads and connects to the prostate, indicating direct invasion (arrowheads). Arrow may indicate fistulation.

Contrast CT showed a lobulation-like tumor with high density in the left pelvic cavity between the prostate and rectum (**Fig. 2A**). The tumor was connected to the prostate with a vague border, indicating direct tumoral invasion. Additionally, metastases of the bilateral lymph nodes were also suspected (**Fig. 2B**). A sagittal image showed that the mass was in contact with the urethra in the prostate, implying fistulation between the rectum and urinary system via the tumor (**Fig. 2C**). T2-weighted MRI indicated that the tumor had a low-intensity mass (**Fig. 3A**). The tumor was connected to the prostate and had invaded the inner gland beyond the outer lesion (**Fig. 3B**). A diffusion-weighted image showed the tumor as a hyperintense mass as well as a right-sided lateral lymph node (**Fig. 3A**). The mass reached the rectum and prostate with possible invasion and fistulation (**Fig. 3C** and **3D**). Cystoscopy was performed to detect the breakage of the urethra to determine whether fistulation had occurred.

**Fig. 4:**
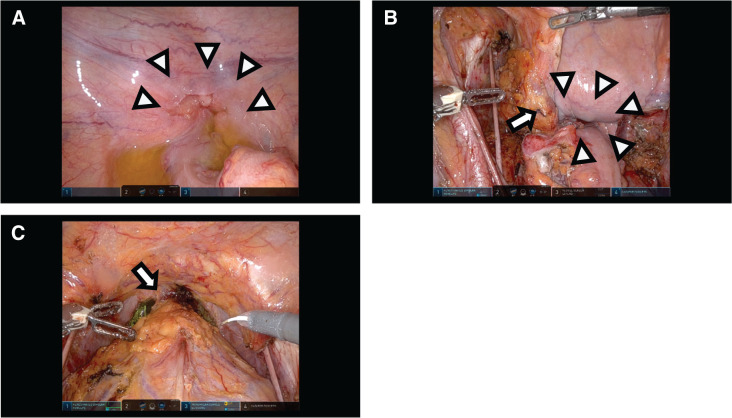
Surgical finding. (**A**) The main tumor is recognized as a deformation of the bottom of the pelvic cavity, indicating direct invasion to the seminal vesicle (arrowheads). (**B**) The image shows left lateral lymph nodes dissection was completed (arrow). The left obturator nerve was preserved. Arrowheads indicate the tissue containing the main tumor inside, meaning that the surface of the cancer was not exposed and prevented cyst rupture. (**C**) The main tumor around the rectum has been dissected with the bladder, prostate, ureters, and seminal vesicle. Bilateral lymph node dissections have also been completed. Arrow indicates the urethra remaining, which would be dissected, followed by tumor removal.

Based on these findings, the patient was diagnosed with rectal SCC without obvious distant metastasis (cT4bN3M0 Stage IIIC). Meanwhile, there was still some possibility of urinary system–derived SCC. He did not want to receive CRT, which is considered the standard initial strategy for rectal and anal SCC; therefore, surgical management was performed for both cure and diagnosis. Under robotic assistance that allow precise manipulations in narrow pelvic space, TPE with bilateral lymph node dissection was performed with multiple organ resection, including the rectum, prostate, seminal vesicles, and urinary bladder (**Fig. 4A** and **4B**). Colostomy and ileal conduit were constructed in his left and right abdomen, respectively. The operative time was approximately 949 min, and intraoperative blood loss was approximately 250 mL.

**Fig. 5:**
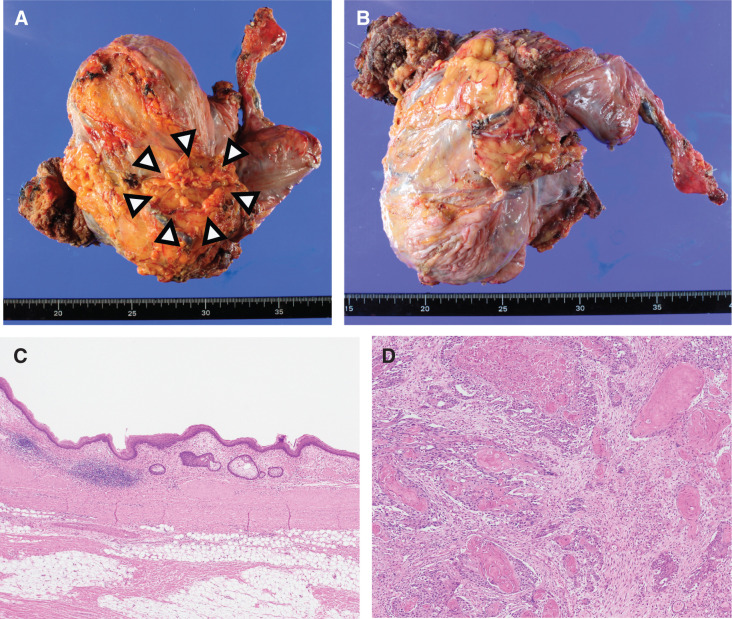
Postoperative pathological findings. (**A** and **B**) Macro findings. The resected specimen showed a tumor composed of a 60-mm cyst and a 40-mm solid lesion between the urinary bladder and upper rectum, attached to the prostate and seminal vesicle (arrowheads). (**C**) The non-tumorous cyst wall is covered by a stratified squamous epithelium with a granular layer and keratinization. Well-differentiated sebaceous glands are observed. Smooth muscle bundles are observed around the cyst wall (HE, × 20). (**D**) Tumor portion. Atypical squamous epithelial cells proliferate invasively forming irregular nests with abnormal keratinization (HE, × 100). HE, hematoxylin and eosin

Macroscopic examination of the resected specimen showed a tumor composed of a 60-mm cyst and a 40-mm solid lesion between the urinary bladder and upper rectum, attached to the prostate and seminal vesicle (**Fig. 5A** and **5B**). The tumor contained various particles that resembled sand-like granules. Microscopic examination revealed a unilocular cyst surrounded by stratified squamous epithelium with keratinization and a granular layer. A small number of sebaceous glands and sweat glands were observed in the cyst wall, and smooth muscle was present around the cyst (**Fig. 5C**). No other teratomatous components were observed. Based on these histological findings, the cyst was diagnosed as a DC. The tumor was continuous with the cyst and was characterized as well-to-moderately differentiated SCC with high keratinization (**Fig. 5D**). The tumor also contained necrotic areas and a foreign body reaction to keratinization. Furthermore, it had directly invaded the seminal vesicle. The DC was located between the bladder and rectum, adjacent to the prostate and seminal vesicles. The SCC arising from the DC had invaded and perforated the rectal mucosa, resulting in partial continuity between the cyst and rectum.

The patient developed adhesive intestinal obstruction at POD 6; however, he was successfully treated using a trans-nasal decompression tube only. The patient was discharged with no other clinical events 39 days after surgery. No recurrence was observed in the first follow-up CT 3 months after surgery.

## DISCUSSION

According to Hawkins and Jackman, congenital cystic tumors caused by a developmental error in the embryonal phase are defined as developmental cysts, and are pathologically classified into epidermoid, dermoid, or tailgut cysts.^1)^ Developmental cysts mostly develop in middle-aged women and in the retrorectal space because the three germ layers in this region are associated with fetal development.^3,4)^ This unusual location and slow growth result in DCs remaining asymptomatic unless they become malignant.^4)^ Although the incidence of malignant tumors arising from a tailgut cyst is 26.6%, the incidence from DCs was reported to be a maximum of 1.8%.^5,6)^ The underlying mechanisms involved in the transformation of developmental cysts to malignancy remain unclear, although chronic inflammatory responses to repeated cyst ruptures as well as genetic mutations, including *TP53* and *PTEN* have been suggested.^7,8)^ However, most cases of DC-derived SCC have been reported in the brain and ovary.^9,10)^ Regarding the pelvic cavity, especially around the rectum, there have been several reports of epidermal cyst–derived SCC in the presacral space, which is extremely rare.^11–13)^ To the best of our knowledge, the current case is the first reported patient with SCC that originated from a DC in the anterior rectal area (i.e., across the presacral space), suggesting that urorectal septum malformation may be related to the oncogenesis.^14)^

The use of CT and MRI, as well as endoscopic examination, are important for the diagnosis of developmental cysts and related malignant diseases. DCs contain desquamated debris, cholesterol, keratin, and water, which may explain the extremely high and low density mass observed by CT of the current case.^15)^ The tumor from our case showed low- and high-intensity signals by T1-weighted and T2-weight MRI images, respectively, and direct tumoral invasion to the urinary system was suspected, suggesting that TPE was a suitable procedure for complete resection. These preoperative findings were confirmed by pathological examination of the resected sample. Thus, MRI can be used to predict the precise tumor margin to be dissected in cases of DC-derived tumors.

Preoperative biopsy is not recommended for patients with epidermal cysts because of the risk of tumor dissemination, abscesses, fecal fistulas, or meningitis.^11)^ Although preoperative diagnosis of a developmental cyst is presumably difficult, the current case had already been diagnosed with SCC by endoscopic biopsy. Nevertheless, DC–derived cancer was not considered until surgery was performed. Accordingly, if pelvic DC–derived SCC is suspected preoperatively, complete surgical excision may be recommended initially as a diagnostic treatment. In this regard, highly precise management is required for DC treatment to avoid cancer dissemination by rupture during surgery and to prevent severe postoperative complications. The patient was young male so that preserving pelvic nerves as long as possible was also important. Therefore, sophisticated robot-assisted surgery is an ideal technique for the management of DC diagnosis.^16)^ In addition, trans-perineal manipulation was simultaneously performed with laparoscopy, in which 2 surgery teams cooperated to see the tumor from both directions, resulting in avoiding cyst rupture. Of note, this is the first report of the surgical resection of DC-derived SCC by robot-assisted TPE with simultaneous bilateral lymph node dissection at the time of writing.

Is CRT an alternative adaptation for pelvic developmental cyst–derived SCC? As mentioned above, CRT is considered the standard therapy for rectal and anal SCC, whereas multimodal therapy combined with surgery is essential for urinary system–derived SCC because of its high malignant potential.^17)^ Some studies have reported the use of radiotherapy for intracranial epidermoid cyst–derived SCC; however, this non-operative management including chemotherapy appears to be less effective than surgery.^18–20)^ Furthermore, no study has reported pelvic DCs. Thus, the recommended strategy for intra-abdominal DC–originated SCC has not yet been elucidated. Because of the difficulty in performing a preoperative diagnosis of DC–derived cancer, diagnostic excision may be initiated in similar cases. In the recent case, whether the cancer was generated from the rectum or urinary system was not clear until surgery. Taken together, we consider that primary surgery should be a validated strategy for pelvic DC–derived SCC if complete resection is possible, and radiotherapy and/or chemotherapy may be performed for unresectable cases.

Developmental cyst–derived SCCs are rare, but might have highly recurrent potential after surgery.^21)^ Accordingly, there is an urgent need for a definitive policy on adjuvant and recurrent treatment. In our case, perirectal lymph node metastasis was recognized synchronously with tumor invasion into the urinary system, indicating that our patient was at high risk for recurrence. Therefore, he was treated with pyrimidine fluoride drugs as adjuvant therapy, resulting in no recurrence to date. This oral anticancer agent was a prodrug of 5-FU that is widely administrated in esophageal or skin SCC. We also plan to take CT every 3 months as well as checking the blood tumor marker level; however, the patient requires long-term follow-up to determine whether relapse occurs.

## CONCLUSIONS

Pelvic DCs and subsequent SCC originating from DCs are extremely rare; thus, further data are required to establish standard diagnostic and treatment strategies. Because of its malignant potential, thorough preoperative imaging evaluation and complete resection using an accurate technique are required for such cases.

## References

[1] Hawkins WJ, Jackman RJ. Developmental cysts as a source of perianal abscesses, sinuses and fistulas. Am J Surg 1953; 86: 678–83. 13104773 10.1016/0002-9610(53)90377-8

[2] Crissman JD. Adenosquamous and squamous cell carcinoma of the colon. Am J Surg Pathol 1978; 2: 47–54. 637188 10.1097/00000478-197803000-00006

[3] Jarboui S, Jarraya H, Mihoub MB, et al. Retrorectal cystic hamartoma associated with malignant disease. Can J Surg 2008; 51: E115–6. 19057717 PMC2592572

[4] Brown IS, Sokolova A, Rosty C, et al. Cystic lesions of the retrorectal space. Histopathology 2023; 82: 232–41. 35962741 10.1111/his.14769

[5] Nicoll K, Bartrop C, Walsh S, et al. Malignant transformation of tailgut cysts is significantly higher than previously reported: systematic review of cases in the literature. Colorectal Dis 2019; 21: 869–78. 30932326 10.1111/codi.14628

[6] Peterson WF. Malignant degeneration of benign cystic teratomas of the overy. A collective review of the literature. Obstet Gynecol Surv 1957; 12: 793–830. 13493921 10.1097/00006254-195712000-00001

[7] Abramson RC, Morawetz RB, Schlitt M. Multiple complications from an intracranial epidermoid cyst: case report and literature review. Neurosurgery 1989; 24: 574–8. 2651960 10.1227/00006123-198904000-00014

[8] Harary PM, Hori YS, Kassu R, et al. Paired molecular profiling of malignant transformation of an epidermoid cyst for potential genetic drivers: illustrative case. J Neurosurg Case Lessons 2025; 9: CASE24849. 40324326 10.3171/CASE24849 PMC12051990

[9] Donofrio CA, Bertazzoni G, Riccio L, et al. Intrasellar dermoid cyst: case report of a rare lesion and systematic literature review comparing intrasellar, suprasellar, and parasellar locations. World Neurosurg 2024; 182: 83–90. 37995988 10.1016/j.wneu.2023.11.057

[10] Causa Andrieu PI, Wahab SA, Nougaret S, et al. Ovarian cancer during pregnancy. Abdom Radiol (NY) 2023; 48: 1694–708. 36538079 10.1007/s00261-022-03768-y PMC10627077

[11] Ohsawa M, Kagawa T, Ochiai R, et al. Rare squamous cell carcinoma arising from a presacral epidermoid cyst: a case report. Int J Surg Case Rep 2020; 66: 283–7. 31884265 10.1016/j.ijscr.2019.12.022 PMC6939061

[12] Wu X, Chen C, Yang M, et al. Squamous cell carcinoma malignantly transformed from frequent recurrence of a presacral epidermoid cyst: report of a case. Front Oncol 2020; 10: 458. 32318346 10.3389/fonc.2020.00458 PMC7146309

[13] Yang DM, Kim HC, Lee HL, et al. Squamous cell carcinoma arising from a presacral epidermoid cyst: CT and MR findings. Abdom Imaging 2008; 33: 498–500. 17680300 10.1007/s00261-007-9287-0

[14] Escobar LF, Heiman M, Zimmer D, et al. Urorectal septum malformation sequence: prenatal progression, clinical report, and embryology review. Am J Med Genet A 2007; 143A: 2722–6. 17937427 10.1002/ajmg.a.31925

[15] Hannon J, Subramony C, Scott-Conner CE. Benign retrorectal tumors in adults: the choice of operative approach. Am Surg 1994; 60: 267–72. 8129248

[16] Jayne D, Pigazzi A, Marshall H, et al. Effect of robotic-assisted vs conventional laparoscopic surgery on risk of conversion to open laparotomy among patients undergoing resection for rectal cancer: the ROLARR randomized clinical trial. JAMA 2017; 318: 1569–80. 29067426 10.1001/jama.2017.7219 PMC5818805

[17] Swanson DA, Liles A, Zagars GK. Preoperative irradiation and radical cystectomy for stages T2 and T3 squamous cell carcinoma of the bladder. J Urol 1990; 143: 37–40. 2294257 10.1016/s0022-5347(17)39857-9

[18] Chen Z, Araya M, Onishi H. Proton beam therapy for malignant transformation of intracranial epidermoid cyst. BMJ Case Rep 2019; 12: e229388. 10.1136/bcr-2019-229388 PMC6663167 31320371

[19] Roh TH, Park YS, Park YG, et al. Intracranial squamous cell carcinoma arising in a cerebellopontine angle epidermoid cyst: a case report and literature review. Medicine (Baltimore) 2017; 96: e9423. 29390569 10.1097/MD.0000000000009423 PMC5758271

[20] Zhang X, Zhan A, He D, et al. Epidermoid cyst in atypical intracranial areas transformed to epidermoid carcinoma: a case report. J Int Med Res 2023; 51: 3000605221148146. 36624959 10.1177/03000605221148146 PMC9834806

[21] Kitano D, Komatsu Y, Omori M. A case of oropharyngeal carcinoma accompanying a presacral malignant epidermoid cyst. Cureus 2024; 16: e69841. 39435196 10.7759/cureus.69841 PMC11492551

